# How does agricultural insurance induce farmers to adopt a green lifestyle?

**DOI:** 10.3389/fpsyg.2023.1308300

**Published:** 2023-12-08

**Authors:** Dainan Hou, Xin Wang

**Affiliations:** ^1^School of Business, Minnan Normal University, Zhangzhou, China; ^2^College of Life Science, Longyan University, Longyan, China; ^3^School of Public Policy and Management, Tsinghua University, Beijing, China; ^4^Chinese International College, Dhurakij Pundit University, Bangkok, Thailand

**Keywords:** agricultural insurance, behavior of agricultural households, SOR model, green lifestyle, theoretical framework, behavioral insurance studies

## Abstract

This study is dedicated to exploring and establishing a theoretical framework for the impact of agricultural insurance on farmers’ green lifestyles. By thoroughly analyzing existing literature, this paper reveals the dual pathways through which agricultural insurance influences farmers’ behavior: one affects production activities, and the other affects lifestyle choices. Drawing on the Stimulus-Organism-Response (SOR) theory, the study elaborates in detail the incentive mechanisms of agricultural insurance, farmers’ cognition of green practices, and how these elements work together to guide a transformation in farmer behavior. This process systematically demonstrates the theoretical logic and pathways through which agricultural insurance promotes the transition to green lifestyles among farmers. Additionally, the study provides a preliminary description of the questionnaire design and its role in supplying foundational data for empirical analysis. The paper posits that agricultural insurance harbors an intrinsic mechanism to induce farmers toward greener lifestyles. We anticipate that this research will bring new perspectives to the field of agricultural insurance and contribute new dimensions to the theoretical framework of behavioral insurance.

## 1 Introduction

This study aims to delve deeply into and construct a theoretical system on how agricultural insurance facilitates farmers’ transition to a green lifestyle. Since the Industrial Revolution, the remarkable enhancement in the level of global industrialization has greatly diversified industrial products and profoundly facilitated people’s lives. However, the production and consumption of industrial products have also brought about a series of environmental issues. These issues are mainly reflected in two aspects: firstly, pollution during the production process, such as the discharge of wastewater, exhaust gases, and waste residue, as well as the residue issues of pesticides and chemical fertilizers in agricultural production; secondly, pollution generated in daily life, including the treatment and discharge of garbage and sewage. The presence of these environmental issues poses a severe challenge to the natural environment on which we depend and requires us to take effective measures to respond to and manage them. According to the “WMO Greenhouse Gas Bulletin” released by the World Meteorological Organization (WMO) on October 26, 2022, the concentrations of three key greenhouse gases - carbon dioxide, methane, and nitrous oxide - reached historical highs in 2021. Notably, the growth rate of carbon dioxide between 2020 and 2021 exceeded the average annual growth rate of the past decade (WMO, 2022). Moreover, the “IPCC 2023: Synthesis Report” clearly states: “Human activities have had a clear impact on the warming of the atmosphere, oceans, and land” (Lee et al., 2023). At present, the governance of global environmental issues has become a consensus in the international community, with countries actively exploring paths for environmental governance and green transformation. The Chinese government attaches great importance to this matter. Since the 18th National Congress of the Communist Party of China, the concept of “building a socialist ecological civilization under the leadership of the Chinese Communist Party” has been incorporated into the Party’s constitution, clarifying the guiding status of ecological civilization construction. The revision of the Environmental Protection Law in 2014 strengthened the legal system for environmental protection; the implementation opinions of the Ministry of Environmental Protection in 2015 accelerated the greening of lifestyles; in 2018, the concepts of “ecological civilization” and “beautiful China” were incorporated into the Constitution, marking that the construction of ecological civilization has been elevated to the level of the country’s fundamental law. In 2020, China announced its goals of achieving carbon peak before 2030 and carbon neutrality before 2060, demonstrating its sense of responsibility in global climate governance. In 2021, the guiding opinions issued by the State Council not only proposed a specific timetable but also outlined a vision for the transformation toward green production and lifestyles. Although China has made significant progress in green development, pollution prevention and control, and ecological protection, there are still many challenges in the green transformation of production and lifestyles. This requires China to continuously innovate in policies and technology to achieve a broader green transformation. Data shows that greenhouse gas emissions caused by household consumption in China have accounted for 52% of the country’s total emissions (“Energy Cold Knowledge|More than Half of the Country’s Carbon Emissions Actually Come from It,” 2020; Ritchie et al., 2020). The transition of residents to a green lifestyle has become an urgent necessity. According to data released by the National Bureau of Statistics of China in 2021, there are approximately 509.79 million farmers in China, accounting for 36.11% of the total population. Their lifestyle has a profound impact on China’s ecological environment. The transformation to a green lifestyle in rural areas is not only an important practice for promoting sustainable development in the new era but also a key to achieving the goal of ecologically livable villages in the rural revitalization strategy. Effectively promoting the transformation to a green lifestyle in rural areas is of great significance for improving the quality of the rural ecological environment, advancing green agricultural development, and also lays a solid foundation for promoting public environmental awareness and constructing an ecological civilization society. Consequently, green transition issues have been given high priority by countries worldwide. Currently, most research focuses on the green transition of production methods, while discussions on lifestyle green transition are relatively scarce (Cao and Gao, 2021). Studies also indicate that between 2000 and 2018, both the carbon emissions produced by rural residents’ lives and the *per capita* carbon emissions have risen sharply. Specifically, the carbon emissions from rural residents’ lives account for 3.0–4.0% of the country’s total emissions (Zhang et al., 2021). Therefore, guiding the transition of lifestyles toward green is of paramount significance for reducing carbon emissions, achieving peak carbon levels, and realizing harmonious coexistence between humans and nature.

Insurance, as a crucial financial service tool, plays an essential role in promoting the transformation of production toward a green and low-carbon model. Agricultural insurance, as an important mechanism for diversifying agricultural risks, has been widely adopted in many countries. Since China began implementing agricultural insurance premium subsidy policies in 2007, the development of agricultural insurance domestically has been impressive. By 2022, the total amount of agricultural insurance in China reached 1,192 billion yuan, providing risk protection totaling 5.46 trillion yuan for nearly 167 million farmer-times. With the continuous expansion of agricultural insurance coverage, it has become an indispensable part of the rural financial service system (Hua and Yang, 2023). Agricultural insurance has extensive coverage in rural China, and it essentially falls within the category of green insurance, playing a role in carbon sink projects. This form of insurance not only promotes the sustainable development of agriculture but also makes a significant contribution to achieving China’s goals of carbon peaking and carbon neutrality (Tuo, 2023). Although existing research has extensively explored how agricultural insurance influences farmer production behavior, such as its impact on production scale (Kim et al., 2020), the use of chemicals in agricultural production (Cai et al., 2021; Li et al., 2022), and the adoption of new technologies (Salazar et al., 2019; Fang et al., 2021; Kumar and Babu, 2021), there is still no consensus. Specifically, studies on how agricultural insurance affects the use of chemicals, which are often used to assess its role in green agricultural production, are particularly noteworthy. Researchers have yet to reach a unified view on this topic: some scholars believe that agricultural insurance may reduce the use of chemicals, while others hold the opposite opinion. These studies undeniably show that agricultural insurance has a certain impact on farmer production behavior, but does it influence their lifestyles? This question piqued our interest. This study aims to explore, from a theoretical perspective, how agricultural insurance impacts the green transition of farmers’ lifestyles and to deeply analyze its intrinsic mechanisms and actual effects.

This paper offers three potential contributions:

Firstly, this study delves deeply into and refines the theoretical framework of interdisciplinary research encompassing “Agricultural Insurance + Behavioral Science + Psychology.” By integrating theories and practices from multiple disciplines, we successfully merged perspectives from agricultural insurance, farmer behavior studies, behavioral psychology, and agricultural technology adoption behavior. This fusion offers the academic community a novel research paradigm.

Secondly, this research introduces the SOR model for the first time, constructing a brand-new analytical framework for the “Agricultural Insurance - Farmer Green Lifestyle Impact Mechanism.” Although existing literature has empirically analyzed the impact of agricultural insurance on farmers’ green agricultural production behavior, the exploration of the underlying behavioral decision-making process remains scant. Not only does this paper discuss in-depth the multifaceted influences of agricultural insurance on agricultural production, but it also deciphers the decision-making logic and process by introducing the SOR model, aiming to unveil the underlying theoretical mechanisms.

Furthermore, to facilitate a more comprehensive empirical analysis, based on the SOR model, we designed a detailed survey questionnaire comprising 43 questions. This aims to capture the shifts in farmers’ personal perceptions and their potential impact on behavior.

The structure of the remaining sections of this paper is as follows: Section 2 provides a literature review, Section 3 delves into mechanism analysis, Section 4 describes the questionnaire design, and Section 5 outlines the forthcoming research content.

## 2 Literature review

Agricultural insurance, as a critically vital mechanism for agricultural risk management, has been broadly applied and promoted on a global scale. A plethora of academic studies have unveiled the profound impact of agricultural insurance on farmers’ anticipated profits (Lusk, 2017). More notably, the presence and implementation of agricultural insurance might further shape and modify farmers’ economic behaviors and decision-making methods (Ma et al., 2021). In light of this, the current study aims to undertake an in-depth literature review on how agricultural insurance influences farmer behavior, aspiring to offer a more systematic and holistic theoretical framework for research in this field. Existing research focuses on exploring how agricultural insurance affects farmers’ production behaviors and production consciousness. The impact on production behavior involves aspects such as the use of agricultural chemicals, decision-making on the scale of agricultural production, and the adoption of new agricultural technologies. As for production consciousness, the studies mainly examine whether agricultural insurance may stimulate moral hazard issues among farmers.

Firstly, regarding the impact of agricultural insurance on farmers’ production behavior: the academic community has conducted extensive and in-depth research on the relationship between agricultural insurance and the input of agricultural chemicals. However, current literature on this issue does not present a unified conclusion. For instance, Babcock and Hennessy (1996) indicated that farmers who purchase agricultural insurance might reduce the use of nitrogen fertilizers in agricultural production. Conversely, Horowitz and Lichtenberg (1993) demonstrated that, compared to uninsured farmers, those who have insurance significantly increased their usage of nitrogen fertilizers, pesticides, herbicides, and insecticides by 19, 21, 7, and 63%, respectively, per acre. Furthermore, He et al. (2020) discovered that yield-based agricultural insurance considerably increased chemical inputs (like total expenditure on fertilizers and other chemicals) while studying agricultural production in the Philippines. This finding suggests that the positive marginal incentive effect evidently outweighs the negative influences of moral hazard. Correspondingly, empirical research by Möhring et al. (2020) in a European agricultural context also affirmed a statistically significant positive correlation between agricultural insurance and pesticide usage, further suggesting that without the presence of agricultural insurance, expenditure on pesticides might potentially decline by 6 to 11%. The influence of agricultural insurance on arable land area and its conservation has also been a focal point in academic research. Goodwin et al. (2004) revealed that, under specific environmental conditions, increasing participation rates in agricultural insurance programs would trigger statistically significant changes in cultivated land area, although these changes remain relatively mild across various observed scenarios. More precisely, in their most extreme case studied, a 30% reduction in premiums due to increased insurance subsidies resulted in an increase in insured land area by 0.2 to 1.1%. Regarding land conservation, Claassen et al. (2011), while studying the transition from grassland to cropland in the North American plains, found that factors like crop insurance, disaster assistance, and market loans collectively led to approximately a 2.9% increase in cultivated land area from 1998 to 2007. Regarding the potential impact of agricultural insurance on the willingness to adopt new technologies. Wei et al. (2021), in their empirical study in Shandong Province, China, found that crop insurance has a significant influence on farmers’ willingness to embrace new technologies, specifically manifesting in three aspects: motivation, capability, and opportunity. It’s worth noting that although agricultural insurance can stimulate farmers’ adoption motivation, this positive stimulatory effect might weaken as farmers’ capabilities enhance.

Secondly, the academic community has undertaken a profound exploration of the issue of moral hazard present in agricultural insurance. Through theoretical and empirical analysis, scholars have confirmed the existence of moral hazard in agricultural insurance. Wu et al. (2020) confirmed the phenomenon of moral hazard in agricultural insurance through empirical research. Further, Suchato et al. (2022) unveiled potential moral hazard behavior in farmers’ irrigation decisions under crop insurance policies.

Upon synthesizing existing literature, we recognize that prior research has laid a solid theoretical foundation and empirical methodology for exploration in this field. However, the current body of literature still shows deficiencies, primarily in the following two research directions that urgently need deepening. Firstly, there is a lack of research on theoretical mechanisms. Regarding the specific theoretical mechanisms of how agricultural insurance induces and influences farmer behavior, current research is still in its nascent stages. While multi-angle and multi-dimensional empirical analyses provide us with evidence of the impact of agricultural insurance on farmer behavior, discussions about its underlying deeper mechanisms are relatively scarce. This article delves into the theoretical mechanisms of agricultural insurance’s impact on farmers’ behavior, aiming to unveil and decode the intrinsic logic of this influence process in order to break open the “black box” of the underlying mechanisms at work.

Secondly, while current studies focus on farmers’ production behaviors, they often overlook the non-production, lifestyle behaviors of farmers. Given the impact of agricultural insurance on farmers’ production behaviors and the potential moral hazard phenomenon therein, we have reason to question: could agricultural insurance exert some influence on the psychological activities of farmers, thereby further affecting their non-production behaviors? Therefore, this paper draws on psychological theory to explore the psychological activities of farmers triggered by the implementation of agricultural insurance and its impact on their lifestyles.

Therefore, this research decides to delve deeper into both theoretical and empirical exploration from two aspects. Firstly, we will introduce the SOR model from the field of psychology, aiming to enhance our understanding of the theoretical mechanisms by which agricultural insurance triggers farmer behavior. Secondly, we will further investigate the potential impact of agricultural insurance on farmers’ non-production behaviors.

## 3 Research findings

### 3.1 Two paths of agricultural insurance’s impact on farmer behavior

**Figure 1:**
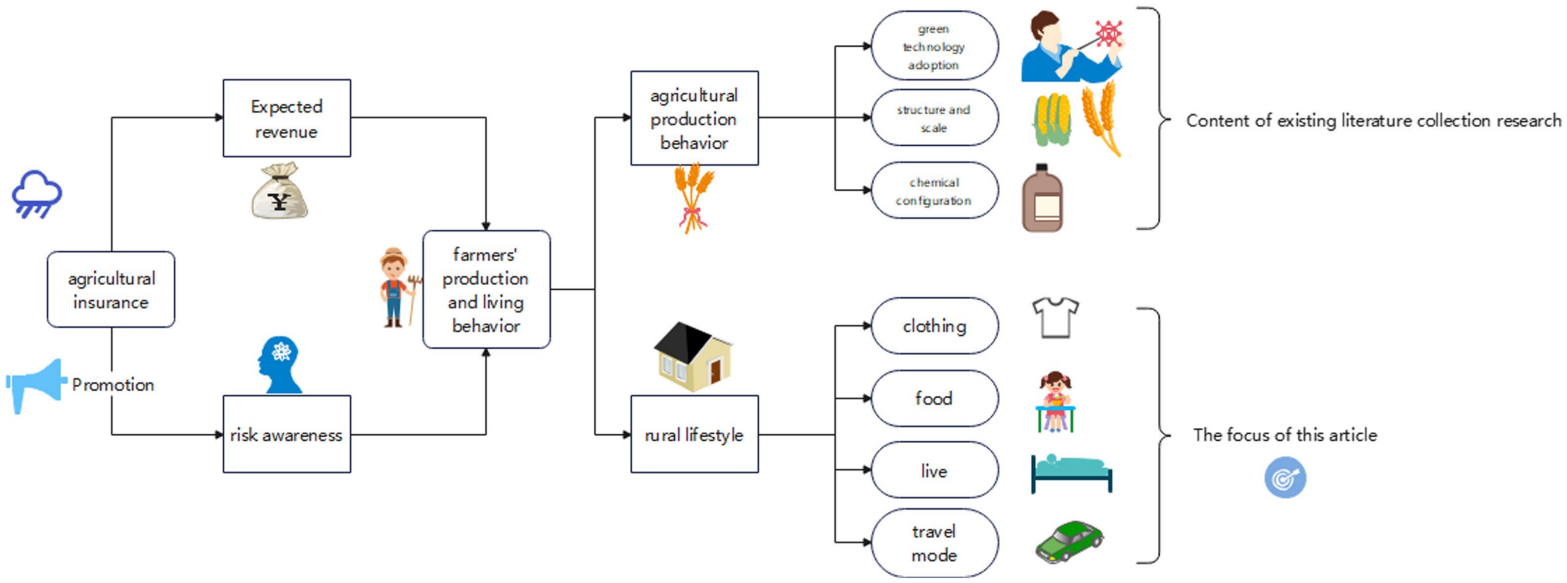
Impact mechanism of agricultural insurance development on farmers’ production and lifestyle behavior.

Agricultural insurance, as a crucial tool for agricultural risk management, not only adjusts farmers’ judgments regarding anticipated production income but also, through promotional and outreach activities, shapes and reinforces farmers’ risk awareness. This profoundly impacts their daily behavioral choices, as illustrated in Figure 1. Based on existing literature and the theoretical framework, this study categorizes the impact of agricultural insurance on farmer behavior into two major paths.

Firstly, there is the direct impact of agricultural insurance on farmers’ production behavior. Reviewing the academic achievements, we observe that scholars have systematically explored this from perspectives like technology adoption, production scale, and chemical inputs, and a detailed summary is provided in the literature review section of this paper. Hence, we can conclude that the core impact of agricultural insurance on farmers’ production behavior mainly revolves around the adoption of new technologies, adjustments in production scale, and choices in chemical inputs.

Secondly, agricultural insurance might indirectly influence farmers’ lifestyles. While existing studies have verified the evident impact of agricultural insurance on farmers’ production behaviors and unveiled potential moral hazard issues during the implementation of agricultural insurance, we theoretically infer that the widespread implementation of agricultural insurance might also trigger changes in farmers’ lifestyle behaviors. Such changes might manifest specifically in aspects of farmers’ clothing, food, housing, and transportation.

### 3.2 Mechanism analysis of agricultural insurance’s impact on rural green lifestyles

#### 3.2.1 The connotation of green lifestyle

The essence of a green lifestyle differs fundamentally from that of green production. The green lifestyle emphasizes harmonious coexistence between humans and nature, aiming to protect the natural environment to the greatest extent while satisfying the demands of human daily activities. Its core philosophy is to weigh and meet various human societal demands based on full consideration of the environmental resource-bearing capacity (Chen and Hou, 2022), striving to achieve the dual goals of protecting natural resources and promoting sustainable economic and societal development (Zheng et al., 2023). Specifically, the green lifestyle mainly focuses on people’s everyday life, encompassing aspects such as the greening of clothing, food, housing, and transportation, green travel, green consumption, and other daily environmental-friendly behaviors, like waste sorting. Additionally, by advocating for the public’s use of environmentally friendly products, participating in green volunteer services, and establishing concepts of green growth and collective construction and sharing (Li and Zhang, 2023), we hope to make green consumption (Sony and Ferguson, 2017), green travel, and green living (Binder and Blankenberg, 2017) the daily choices for the majority of the public. Based on existing literature definitions and descriptions of the green lifestyle, this study will delve deeply into the green living practices of rural residents, focusing on the four aspects of clothing, food, housing, and transportation.

#### 3.2.2 Stimulus-organism-response model

The stimulus-organism-response (SOR) model was initially proposed by the American psychologist Woodworth and gradually matured through further development and refinement by researchers like Mehrabian. Originating from the behaviorist psychology’s SR (Stimulus–Response) model, it has evolved into a theoretical framework widely used to analyze individual behavioral responses under the influence of internal emotions and external environmental stimuli (Ding and Zhang, 2022; Sun, 2022). The SOR model primarily consists of three components: “Stimulus” (S), “Organism” (O), and “Response” (R). Here, the Stimulus (S) represents influencing factors from the external environment, Organism (O) describes the individual’s internal psychological reaction upon receiving external stimuli, and Response (R) manifests as the specific behavior exhibited by the individual after receiving the stimuli.

In this study, we utilize the SOR model to dissect the potential mechanisms through which agricultural insurance induces farmers to adopt a green lifestyle. Specifically, this paper constructs a theoretical model that reveals the mechanisms by which agricultural insurance affects farmers’ behavior, based on the framework of the Stimulus-Organism-Response (SOR) model. Specifically, the “Stimulus” (S) here primarily refers to the promotional activities of agricultural insurance and various related information disseminated during its implementation process; “Organism” (O) points to the psychological perceptions and cognition of farmers upon receiving this agricultural insurance-related information; and “Response” (R) specifically refers to the green lifestyle practices adopted by farmers, encompassing but not limited to specifics in clothing, food, housing, and transportation.

#### 3.2.3 Analysis of the mechanism of influence

Based on the theoretical framework of the SOR model, the entire cycle of agricultural insurance encompasses several main stages: pre-sales promotional activities, risk protection provided during sales, disaster prevention strategies implemented by insurance companies, and post-disaster claims services. Within this cycle, farmers, as the key target audience for agricultural insurance, are exposed to a plethora of information related to agricultural insurance. This information can trigger specific perceptions in farmers, leading to subsequent behavioral changes. This study focuses particularly on the potential influence of agricultural insurance-related information on the green lifestyle of farmers. Therefore, the core framework of this study begins with agricultural insurance as the external Stimulus (S), passes through farmers’ perception of a green lifestyle as the Organism (O), and finally explores its terminal impact on the green lifestyle Response (R) of farmers. This paper aims to delve deeply into the intrinsic relationships among agricultural insurance, farmers’ green lifestyle perception, and their green living practices.

First, let us explore the aspect of environmental Stimulus (S). In the pre-sales phase of agricultural insurance, local governments and insurance institutions utilize a combination of traditional and modern promotional channels. For instance, they may employ bulletin boards, agricultural training courses (especially during off-season periods), and digital platforms like WeChat to promote agricultural insurance. In this phase, farmers might actively seek out or passively receive information from the aforementioned sources. As they transition into the sales phase, where insurance is underwritten, professionals from insurance companies and grassroots agricultural departments provide detailed explanations of agricultural insurance products. This includes key terms like insurance liabilities and exclusions. Notably, many insurance products have clauses related to green production, such as land protection standards and environmentally friendly livestock management. This means farmers are exposed to a wealth of green production information during this phase. In the post-sales phase, which includes risk protection, disaster prevention measures, and disaster claims provided by insurance companies, farmers receive further stimuli.

Second, let us focus on the Organism (O) level. According to the theoretical framework of information processing psychology, an individual’s cognitive process can be viewed as an information processing mechanism. In this mechanism, individuals first perceive external stimuli through sensors, then analyze and integrate based on the knowledge stored in their information processing system, and finally output the processed information through effectors (Ji et al., 2023). At the perception stage, farmers process the external information they receive. It’s noteworthy that this process might be influenced by various factors, including but not limited to age, gender, educational background, agricultural operational status, and income level. After considering these factors, this study particularly focuses on trust, perceived usefulness, and emotional arousal as dimensions to describe farmers’ perceptions. Specifically, the trust farmers place in agricultural insurance information, their perceived usefulness of this information, and the emotional reactions elicited by this information can all influence their attitudes and behaviors toward a green lifestyle. It’s worth mentioning that while previous studies have employed advanced measurement tools like electroencephalograms (EEG) and eye-tracking to study individual consumer perceptions and experiences (Su and Lü, 2022; Ren et al., 2023), these methods, due to their high requirements for equipment and experimental environment, are hard to apply in this study. Consequently, we have adopted a questionnaire survey method, drawing inspiration from the PAD psychological scale, to capture subtle shifts in farmers’ perceptions. The detailed design of the questionnaire will be elaborated in the fourth section of this paper.

**Figure 2:**
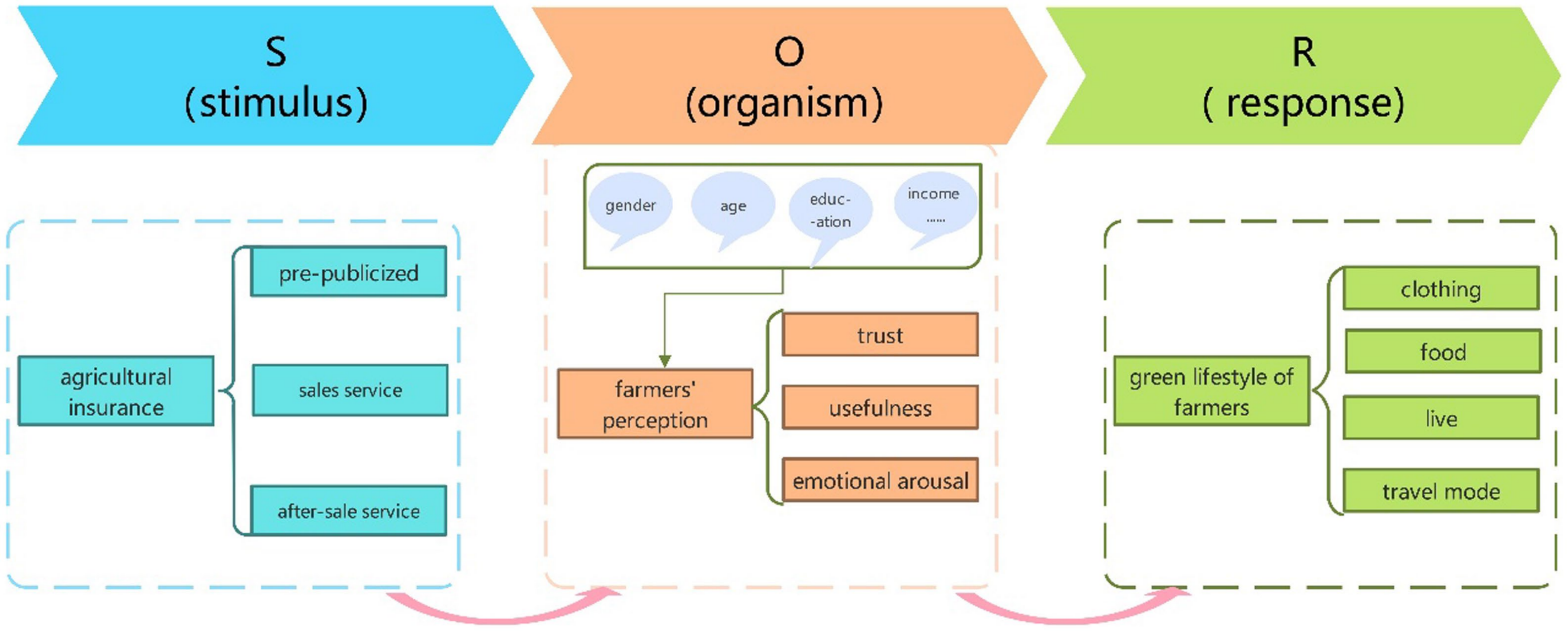
Analysis model based on the SOR theory.

Lastly, let us consider the Response (R) dimension. Upon receiving information stimuli related to green agricultural production through agricultural insurance, farmers’ psychological activities might be affected, leading to changes in their lifestyles. Being the primary audience for agricultural insurance, when farmers receive such green agriculture-related stimuli, their perceptions of this information, especially in terms of trust, perceived usefulness, and emotional arousal, might motivate them to respond more actively and practice the advocated green agricultural suggestions. Specifically, this behavioral change could manifest in their daily lifestyles, covering aspects such as clothing, food, housing, and transportation. The logical relationship of this process is showcased in Figure 2.

## 4 Further discussion

To delve into and validate the intrinsic mechanism of the impact of agricultural insurance on farmers’ green lifestyle, this study meticulously crafted a survey questionnaire. During the design process, we referred to the PAD psychological scale and drew inspiration from classic questionnaire templates in related fields. The questionnaire is divided into four main sections, comprising a total of 43 specific questions.

The first section of the questionnaire focuses on the collection of basic information, aiming to gain an in-depth understanding of the background and general situation of the respondents. This section may influence farmers’ reception and perception of information related to agricultural insurance. This section includes 10 questions, encompassing information such as the respondent’s location, gender, whether they hold village official positions, age, educational background, years engaged in agricultural production, type of agricultural operation, agricultural operational income in 2022 and its proportion in the total income, and whether they have purchased agricultural insurance. It’s worth noting that some of these items, like income level and whether they are village officials, are considered mediator variables in subsequent empirical studies. Other factors, such as location, age, and education level, are considered potential moderator variables.

The second section primarily probes into the influencing factors of environmental Stimulus (S), encompassing four dimensions: pre-sales promotion, insurance products, disaster prevention measures, and claims services, with a total of 12 specific questions. This section collects data on the external stimuli related to agricultural insurance information received by farmers. From this section onward, all questions adopt the Likert five-point scale format. In the pre-sales promotion dimension, the question design centers on the association between agricultural insurance and green development. For instance, it delves into the promotional intensity and strategies of the “agricultural insurance + ecological product” financial model and its actual implementation. In the insurance product dimension, our focus is on how green thinking is integrated into agricultural insurance products and the related green clause design and implementation. For the disaster prevention measures dimension, questions revolve around disaster prevention technologies, especially those emphasizing environmentally friendly applications and promotions. Lastly, in the claims services dimension, we delve into green standards in claims clauses, green technologies used in loss assessments, and how to embody the concept of green service throughout the claims process.

The third section zeroes in on farmers’ green perception (O), covering the three core dimensions of trust, perceived usefulness, and emotional arousal, with a total of 9 questions. This section gathers information on the green perception of farmers after they receive external stimuli related to agricultural insurance. In the trust dimension, we focus on the trust farmers place in the green elements and content involved in agricultural insurance, including their assessment of the authenticity and reliability of this content. For the usefulness dimension, we approach from the farmers’ perspective, deeply understanding their acceptance level of information related to green production in agricultural insurance and how this information assists their daily production activities. The emotional arousal dimension takes a deeper stance, exploring the emotional resonance farmers feel when encountering agricultural insurance-related content. This dimension complements the previous two and delves deeper into the psychological and emotional facets of farmers.

The fourth section studies farmers’ green lifestyle (R), touching upon the four key dimensions of “clothing,” “food,” “housing,” and “transportation,” comprising 12 questions. This section collects information on the specific behaviors of farmers following the “stimulus–response” process. In the “clothing” dimension, we investigate how farmers deal with old clothes, their frequency of purchasing new garments, and the underlying green consumption concepts. For the “food” segment, we start with daily dietary habits, including farmers’ use of disposable tableware, their attitudes toward leftover food, and the scale of large-scale events like weddings and funerals, thus understanding their views on food wastage. In the “housing” dimension, we are interested in how farmers practice green living in their homes, such as whether they choose environmentally friendly interior decoration materials, their waste sorting habits, energy-saving practices, and the use of sanitary toilets. In the “transportation” segment, we mainly explore farmers’ travel choices, especially for different distances, probing the environmental consciousness behind them.

After the questionnaire design was completed, we conducted a small-scale pilot survey and invited experts from related fields for in-depth consultations. Based on feedback from these preliminary efforts, we meticulously revised and optimized the original questionnaire, resulting in the current version. In terms of data analysis and processing, this paper plans to employ statistical methods such as structural equation modeling to explore issues related to impact mechanisms and mediating effects. If peers in academia are interested in this research project, we warmly welcome you to contact us. We will provide the complete questionnaire content and data from future large-scale surveys. We also sincerely hope experts and scholars can offer valuable opinions and suggestions for our research and questionnaire. After collecting more feedback, we plan to refine the questionnaire further and distribute it more widely.

## 5 Conclusion and prospects

### 5.1 Conclusion

This study focuses on exploring the deep-seated theoretical mechanism of how agricultural insurance induces farmers to transition to a green lifestyle. At the outset of the research, we conducted an in-depth exploration of the two main pathways through which agricultural insurance influences farmers’ behavior: production behavior and lifestyle behavior. Furthermore, we clearly defined the concept of a “green lifestyle.” Subsequently, using the SOR model as the core theoretical framework, we elaborated on how agricultural insurance induces farmers to undergo a green transformation in their lifestyles. Through our analysis, we discovered that agricultural insurance not only has an impact on farmers’ production behavior but may also affect their lifestyle behaviors. Additionally, we designed a corresponding survey questionnaire for subsequent empirical research.

### 5.2 Future research directions

To delve deeper into and validate the aforementioned theoretical mechanisms and pathways, and to uncover the latent factors in farmers’ decision-making processes—often referred to as the “black box”—we meticulously crafted a survey questionnaire. In the subsequent research, we plan to revise this questionnaire with greater rigor, initiate its distribution, systematically collect data, and conduct empirical analyses for validation. We believe that the results of the empirical research will provide valuable insights into how external stimuli, like agricultural insurance, can have long-term impacts on farmers’ behavioral choices. Such findings could potentially inform policy-making, aiding in the promotion of green lifestyles in rural areas. By understanding the psychological and emotional factors that mediate these behavioral changes, we can tailor interventions more effectively, leading to more sustainable agricultural practices and lifestyles. Future research could also explore the feedback loop—how adopting a green lifestyle might influence farmers’ perceptions of and trust in agricultural insurance. Understanding this dynamic relationship could provide further insights into how to sustainably promote and integrate green practices in rural communities. In the future, we aim to further explore the mechanisms and pathways through which agricultural insurance influences farmers’ green lifestyles through empirical research. This research will provide important references for the optimization of agricultural insurance policies, product design, and the services of agricultural insurance companies. At the same time, we anticipate that the continuous improvement of agricultural insurance will assist farmers in transitioning to green lifestyles, support China in achieving its proposed “dual carbon” goals, and contribute to the construction of an ecological civilization.

## Data availability statement

The original contributions presented in the study are included in the article/supplementary material, further inquiries can be directed to the corresponding author.

## Ethics statement

Ethical review and approval was not required for the study on human participants in accordance with the local legislation and institutional requirements. Written informed consent from the patients/participants OR patients/participants legal guardian/next of kin was not required to participate in this study in accordance with the national legislation and the institutional requirements.

## Author contributions

DH: Conceptualization, Funding acquisition, Methodology, Project administration, Writing – original draft. XW: Formal analysis, Funding acquisition, Project administration, Writing – review & editing.
